# Glucocorticoid impairs cell-cell communication by autophagy-mediated degradation of connexin 43 in osteocytes

**DOI:** 10.18632/oncotarget.9034

**Published:** 2016-04-27

**Authors:** Junjie Gao, Tak Sum Cheng, An Qin, Nathan J. Pavlos, Tao Wang, Kai Song, Yan Wang, Lianzhi Chen, Lin Zhou, Qing Jiang, Hiroshi Takayanagi, Sheng Yan, Minghao Zheng

**Affiliations:** ^1^ Centre for Orthopaedic Research, School of Surgery, The University of Western Australia, Nedlands, Western Australia, Australia; ^2^ Shanghai Key Laboratory of Orthopedic Implants, Department of Orthopedic Surgery, Shanghai Ninth People's Hospital, Shanghai Jiao Tong University School of Medicine, Shanghai, China; ^3^ Division of Orthopaedic Surgery, Department of Surgery, Guangdong General Hospital, Guangdong Academy of Medical Sciences, Guangzhou, China; ^4^ Key Laboratory of Combined Muti-organ Transplantation, Ministry of Public Health, State Key Laboratory for Diagnosis and Treatment of Infectious Diseases, Division of Hepatobiliary Pancreatic Surgery, First Affiliated Hospital, Zhejiang University School of Medicine, Hangzhou, China; ^5^ Department of Sports Medicine and Adult Reconstruction Surgery, Drum Tower Hospital Affiliated to Medical School of Nanjing University, Nanjing, Jiangsu, China; ^6^ Department of Immunology, Graduate School of Medicine and Faculty of Medicine, The University of Tokyo, Tokyo, Japan; ^7^ State Key Laboratory for Diagnosis and Treatment of Infectious Diseases, Collaborative Innovation Center for Diagnosis and Treatment of Infectious Diseases, The First Affiliated Hospital, College of Medicine, Zhejiang University, Hangzhou, China

**Keywords:** osteocyte, connexin 43, autophagy, glucocorticoid, mTORC1, Gerotarget

## Abstract

Osteocytes comprising over 90% of the bone cell population are highly susceptible to the adverse effects of glucocorticoids (GC) administration. Here we observed that Dexamethasone (Dex) induces a robust cytoskeleton rearrangement and decreases Cx43 protein expression in osteocyte-like MLO-Y4 cells. Using a Dmp1^Cre^-mT/mG osteocyte *ex vivo* culture system, we found significant shortening of dendritic processes in primary osteocytes following Dex administration. Loss of dendritic processes is a consequence of reduced Cx43 connectivity upon Dex induced autophagy in both RFP-GFP-LC3B transfected MLO-Y4 cells and primary calvarial osteocytes from LC3^GFP^ transgenic mice. Upon the induction of autophagy by Dex, Cx43 was internalized into autophagosome/autolysosomes and degraded by autophagy. The degradation was attenuated following lysosomal inhibition using chloroquine (CLQ) and suppression of autophagy by Atg5 silencing. Inhibition Akt-mTORC1 signaling by Dex induces autophagy subsequently resulting in Cx43 degradation. Activation of Akt phosphorylation by IGF-1 attenuated Dex induced autophagy and degradation of Cx43. Together, we demonstrated that GC impair osteocyte cell-cell connectivity via autophagy mediated degradation of Cx43 through inhibition of the Akt-mTORC1 signaling. This may account for the deleterious effect of GC-induced bone loss.

## INTRODUCTION

Being entombed within the mineralized matrix of bone, osteocytes form a complicated yet sophisticated network of intercellular connections *via* their dendritic processes to communicate and regulate neighboring osteocytes and cells on the bone surface. The dendritic processes enable transmission of both chemical and mechanical signals from cell to cell coordinating and initiating the necessary cellular events of bone resorption and formation during bone modeling and remodeling processes. Connexin 43 (Cx43), a major hemichannel protein plays an important role in maintaining dendritic connection between neighboring osteocytes [1, 2]. Cx43 has been shown to play critical roles in bone growth, remodeling, mechanotransduction and survival of osteocytes [3, 4]. Mice with 2.3-kb Col1a1 promoter driven osteoblast/osteocyte-specific deletion of Cx43 display reduced cortical bone thickness and density with expanded bone marrow cavity [5]. Furthermore, loss of Cx43 in osteocytes resulted in decreased sclerostin expression and increased osteocyte apoptosis [5]. In addition, MLO-Y4 osteocyte-like cells that are deficient in Cx43 display an increase in the RANKL/OPG ratio compared to control *in vitro* [6, 7]. Interestingly, chondro-osteogenic lineage Cx43 deficient mice exhibit increased bone resorption and TRAP positive osteoclasts [6, 7]. Together, these data suggested that Cx43 in osteocytes plays a critical role regulating both bone resorption and formation to maintain bone hemostasis [1, 8].

Glucocorticoids (GCs) are important therapeutic agents that have been widely used as anti-inflammatory and immunosuppressive drugs. However, the therapeutic benefits are unfortunately accompanied by serious complications including systemic bone loss, increased risk of fragility fractures and osteonecrosis [9, 10]. Given osteocytes account for more than 90% of the bone cell population, they are highly susceptible to the adverse effect of GC therapy. Although previous study has shown that GC induces osteocyte autophagy, the subsequent implication of osteocyte autophagy remains unclear [11]. In this study, we employed Dmp1^Cre^-mT/mG and LC3^GFP^ transgenic mice to show that GC impairs osteocyte connectivity by inducing autophagy mediated Cx43 degradation both *in vitro* and *ex vivo*. The impairment of osteocyte dendritic network may contribute to the deleterious bone effect associated with GC administration *in vivo*.

## RESULTS

### GC-treatment disrupts the dendritic network of osteocytes

**Figure 1:**
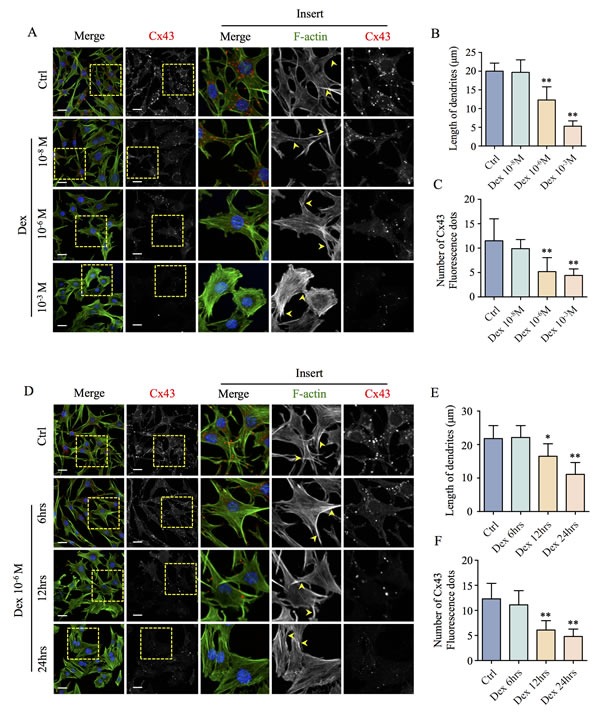
Effects of Dex on MLO-Y4 osteocyte-like cell morphology and Cx43 expression *in vitro* (**A**) Confocal analysis of the dose-dependent decrease in Cx43 expression and morphological rearrangement of actin cytoskeleton of MLO-Y4 cells *in vitro*. MLO-Y4 cells cultured on glass coverslips and treated with various doses of Dex (10^−8^, 10^−6^ and 10^−3^M) were fixed with 4% PFA and stained with rhodamine-conjugated phalloidin (F-actin; green), Cx43 (red), Hoechst 33258 (nuclei; blue), and examined by confocal microscopy. Arrow heads indicate the dendritic processes. The average lengths of dendritic processes and the number of Cx43 fluorescence dots following treatment with indicated concentrations of Dex were quantified (**B** and **C**). (**D**) Time-dependent decrease in Cx43 expression and morphological rearrangement of actin cytoskeleton in MLO-Y4 cells following treatment with 10^−6^M Dex. MLO-Y4 cells cultured on glass coverslips and treated with 10^−6^M Dex for 6, 12, or 24hrs were fixed with 4% PFA and stained with rhodamine-conjugated phalloidin (F-actin; green), Cx43 (red), Hoechst 33258 (nuclei; blue), followed by examination under confocal microscopy. Arrow heads indicate the dendritic processes. The average lengths of dendritic processes and the number of Cx43 fluorescence dots for each time point were quantified (**E** and **F**). All data presented are representative of at least three independent experiments, with quantitative analysis calculated from at least 40 cells in each well. All bar graphs were compared to control and expressed as means ± SD, *p<0.05, **p<0.01. Scale bars = 10μm.

**Figure 2:**
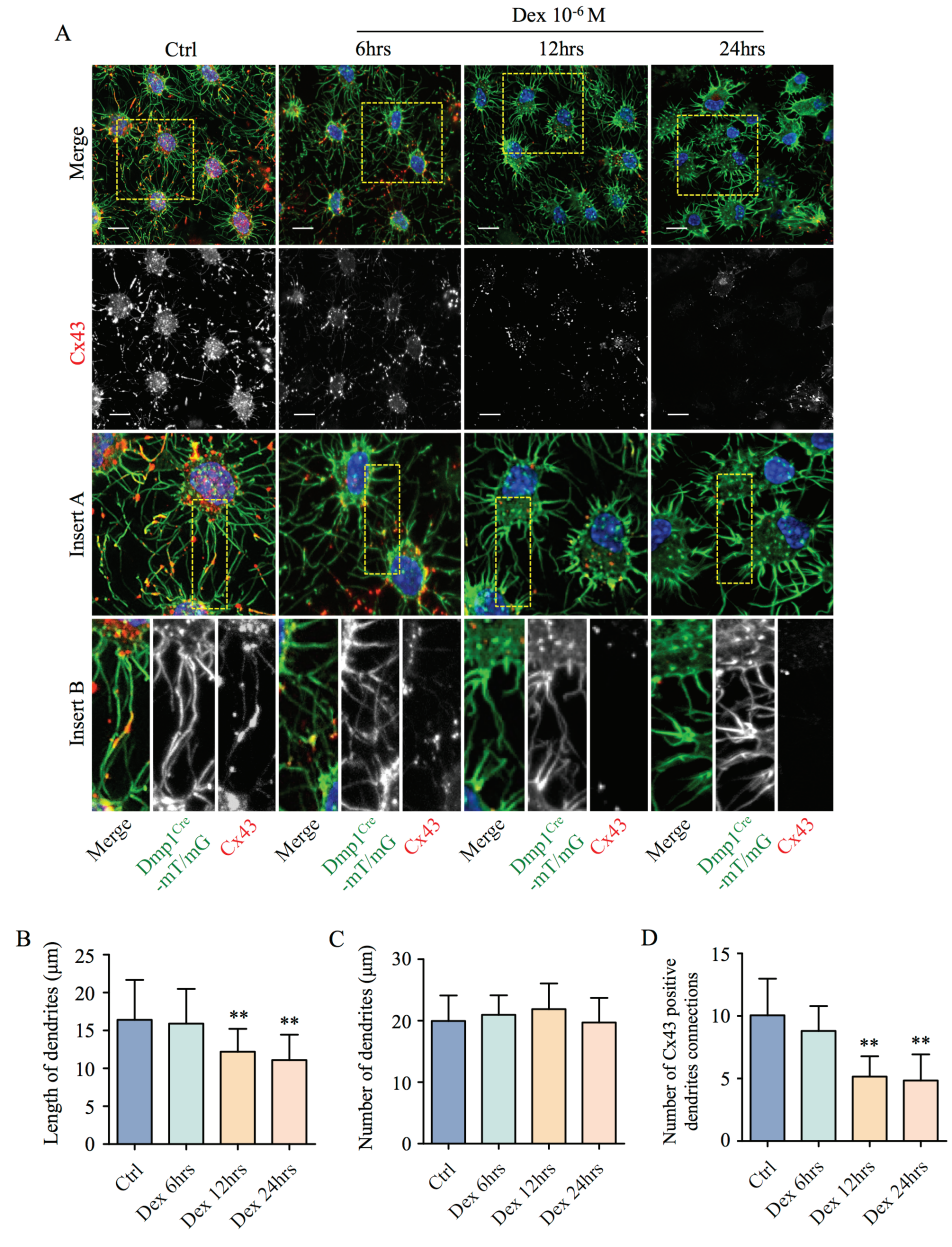
Dex induces time-dependent shortening of dendritic processes, decrease connectivity and reduced expression of Cx43 in primary calvarial osteocytes *ex vivo* (**A**) Time-dependent effects of Dex on DMP1^Cre^-mT/mG primary calvarial osteocytes dendritic network and Cx43 expression and localization were examined by confocal microscopy. Calvariae from Dmp1^Cre^-mT/mG mice treated with 10^−6^M Dex for 6hrs, 12hrs or 24hrs were fixed in 4% PFA for 2hrs at RT following by staining with antibody against Cx43 (red) and Hoechst 33258 (nuclei; blue). Immunoreactivity of membrane-targeted EGFP (mG), Cx43 and Hoechst nuclei staining were examined by confocal microscopy. The average dendritic length (**B**), average number of dendritic processes per cell (**C**), Cx43 localization to dendritic tips (**D**) were quantified for each time point assessed. All data presented are representative of at least three independent experiments, with quantitative analysis calculated from at least 20 cells in each well. All bar graphs were compared to control and expressed as means ± SD, *p<0.05, **p<0.01. Scale bars = 10μm.

The osteocyte dendritic network formed by dendritic connections linked by Cx43 between neighboring osteocytes plays a critical role in bone homeostasis [12]. To examine the effect of GC treatment on osteocyte dendritic connectivity, we first performed *in vitro* analysis on MLO-Y4 osteocyte-like cells. As shown in Figure 1A, untreated MLO-Y4 cells exhibit characteristic dendritic processes and strong Cx43 immunoreactivity in both dendritic processes and cytoplasmic perinuclear region. MLO-Y4 cells treated with Dex demonstrated a dose-dependent shortening of dendritic processes (in a range from 10^−8^M to 10^−3^M), which were accompanied by a drastic rearrangement of actin cytoskeleton characterized by increased stress fiber formation and a significant decrease in Cx43 immunoreactivity from 10^−6^M Dex treatment (Figure 1A, 1B and 1C). Furthermore, MLO-Y4 cells treated with 10^−6^M Dex for time-dependently (in a range from 6hrs, 12hrs to 24hrs) resulted in dendritic shortening and generalized cytoskeletal rearrangement as well as decreased Cx43 expression. (Figure 1D, 1E and 1F). To further confirm whether Dex exerts similar effects on primary osteocytes, we employed *ex vivo* cultured primary calvarial osteocytes from Dmp1^Cre^-mT/mG mice treated with Dex to examine the morphological changes and the expression of Cx43 by confocal microscopy. As shown in Figure 2A, primary calvarial osteocytes from untreated control showed elaborate web-like networks of dendritic connections. Cx43 immunoreactivity was observed in both connective points between osteocytic dendritic processes and in the perinuclear region of individual cells. When cells were treated with Dex at 10^−6^M, we observed a time-dependent shortening of dendrites and the loss of intercellular connectivity without changes in the number of dendrites sprouting from each cell (Figure 2B and 2C). The shortening of the dendrites/loss of connectivity coincided with the decrease in Cx43 immunoreactivity (Figure 2B and 2D), indicating an association between the reduction of dendritic length/connectivity and the decreased expression of Cx43. Together, these observations showed that osteocytes utilize Cx43 hemichannels to form dendritic connections with neighboring osteocytes. Administration of GC, in the form of Dex impairs cell-cell connectivity by inducing shortening of the dendritic processes, reduction in dendritic connections owing to loss of Cx43 expression.

### GC induces Cx43 degradation *via* autophagy in osteocytes

Because previous studies have demonstrated that GC treatment leads to the induction of autophagy in osteocyte-like cells [11]. We reasoned that the loss of Cx43 expression observed in both MLO-Y4 cells and primary osteocytes may reflect autophagy-mediate Cx43 internalization and degradation.

**Figure 3:**
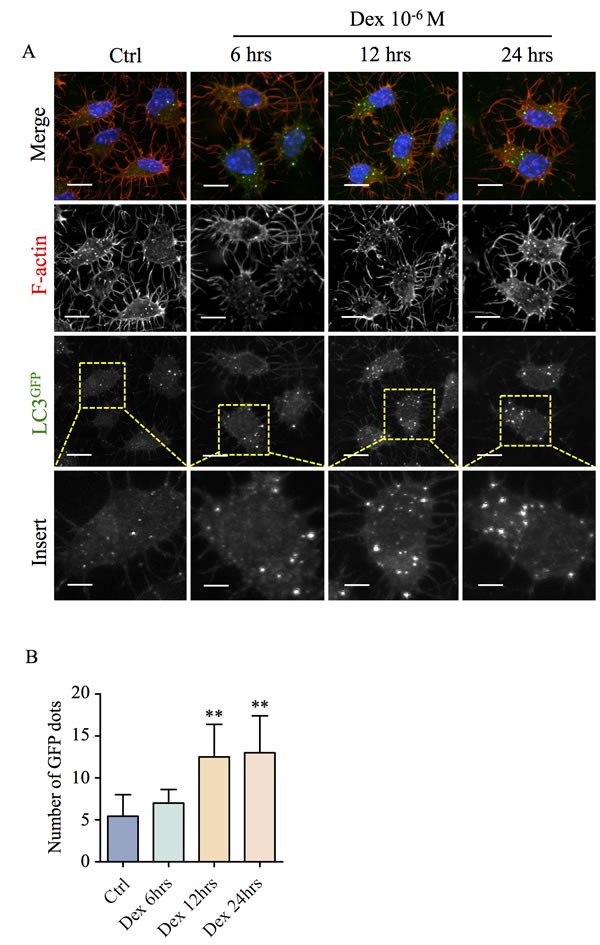
Time-dependent induction of autophagy by Dex in primary calvarial osteocytes *ex vivo* (**A**) Time-dependent accumulation of autophagic vesicles following treatment with Dex in primary calvarial osteocytes *ex vivo*. Primary calvarial osteocytes from LC3^GFP^ mice calvariae were treated with 10^−6^M Dex for 6hrs, 12hrs or 24hrs. Calvariae were then fixed in 4% PFA for 2hrs at RT followed by staining with rhodamine conjugated phalloidin (F-actin; red) and Hoechst 33258 (nuclei; blue). Immunoreactivity and fluorescence of LC3^GFP^ puncta were investigated by confocal microscopy. Scale bars = 10μm. The average number of GFP puncta representing LC3^+ve^ autophagic vesicles were quantified (**B**). All data presented are representative of at least three independent experiments, with quantitative analysis calculated from at least 20 cells in each well. All bar graphs were compared to control and expressed as means ± SD, **p<0.01.

As previously observed [11, 13, 14], treatment of osteocytes with Dex resulted in a dose-dependent increase in autophagasome formation as demonstrated by positive CYTO-ID^®^ staining (Suppl. Figure 1A), as well as the time-dependent conversion and maturation of neutral autophagosomes (GFP; green/yellow) to acidic autolyososomes (RFP; red) indicated by the tandem autophagy sensor, RFP-GFP-LC3B in MLO-Y4 cells *in vitro* (Suppl. Figure 1B). This was further confirmed in our *ex vivo* culture of primary calvarial osteocytes from LC3^GFP^ mice which demonstrated a time-dependent increase in autophagic flux according to the increment in LC3^GFP^ fluorescence puncta following Dex administration (Figure 3A and 3B). Strikingly, the onset of autophagy coincided with decreased Cx43 expression and ensuing dendritic shortening/connectivity. Importantly, this effect is specific to autophagic induction and not related to osteocyte apoptosis as confirmed by (i) flow cytometric analyses for Annexin-V, (ii) lactate dehydrogenase (LDH) cytotoxicity assay (Suppl. Figure 2A and Suppl. Figure 2B) and (iii) assessment of nuclei fragmentation (Suppl. Figure 3).

**Figure 4:**
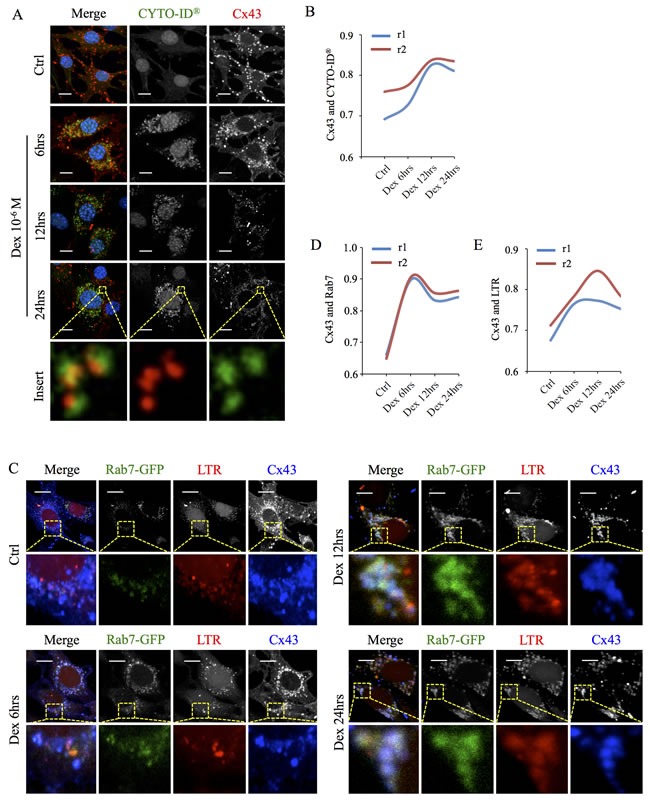
Cx43 is localized to autophagosomes/autolysosomes following Dex treatment (**A**) Time-dependent localization of Cx43 to autophagosome in MLO-Y4 cells treated with Dex *in vitro*. MLO-Y4 cells treated with 10^−6^M Dex for indicated time points were fixed and stained with autophagosome marker CYTO-ID^®^ (LC3; green), Cx43 (red) and Hoechst 33258 (nuclei; blue) and examined under confocal microscopy. Pearson's correlation coefficient (r1) and overlap coefficient (r2) were calculated for CYTO-ID^®^ and Cx43 immunoreactivities (**B**) using JACoP plugin in ImageJ. (**C**) Cx43 is degraded by the autophagosomal/autolysosomal pathway. MLO-Y4 cells transiently transfected with lysosome marker Rab7-GFP (green) and treated with 10^−6^M Dex for indicated times were incubated with LysoTracker Red (LTR; red), fixed and stained for Cx43 (blue). Immunoreactivities were examined under confocal microscopy. Pearson's correlation coefficient (r1) and overlap coefficient (r2) were calculated for Cx43 and Rab7-GFP (**D**), and for Cx43 and LTR (**E**) using JACoP plugin in ImageJ. All data presented are representative of at least three independent experiments, with correlation coefficient analysis conducted for at least 20 cells. Scale bars = 10μm.

To further explore whether autophagy is responsible for Cx43 degradation leading to the decrease in its expression, MLO-Y4 cells treated with 10^−6^M Dex for various times were co-stained for Cx43 and CYTO-ID^®^ (LC3-II). As shown in Figure 4, Dex administration time-dependently induced the formation of cytoplasmic autophagic vesicles that were also positive for Cx43 (Figure 4A). Pearson's coefficient (r1) and overlap coefficient (r2) analyses between CYTO-ID^®^ (LC3-II) and Cx43 immunoreactivity confirmed the significantly positive correlation at 6hrs (r1 = 0.73 and r2 = 0.77) which peaked at 12hrs (r1 = 0.81 and r2 = 0.83) following Dex treatment (Figure 4B). Because autophagy utilizes the lysosomal pathway for protein degradation, we next examined whether the encapsulated Cx43 was delivered to mature autolysosomal vesicular structures for degradation. As shown in Figure 4C, immunofluorescence analysis demonstrated the time-dependent colocalization of Cx43 immunoreactivity with lysosomal markers Rab7-GFP and LysoTracker Red (LTR). Pearson's coefficient and overlap coefficient for the time-dependent colocalization of Cx43 immunoreactivity with Rab7^GFP+ve^ and LTR^+ve^ vesicular structure were r1 = 0.897 and r2 = 0.905, and r1 = 0.773 and r2 = 0.846 respectively (Figure 4D and 4E).

**Figure 5:**
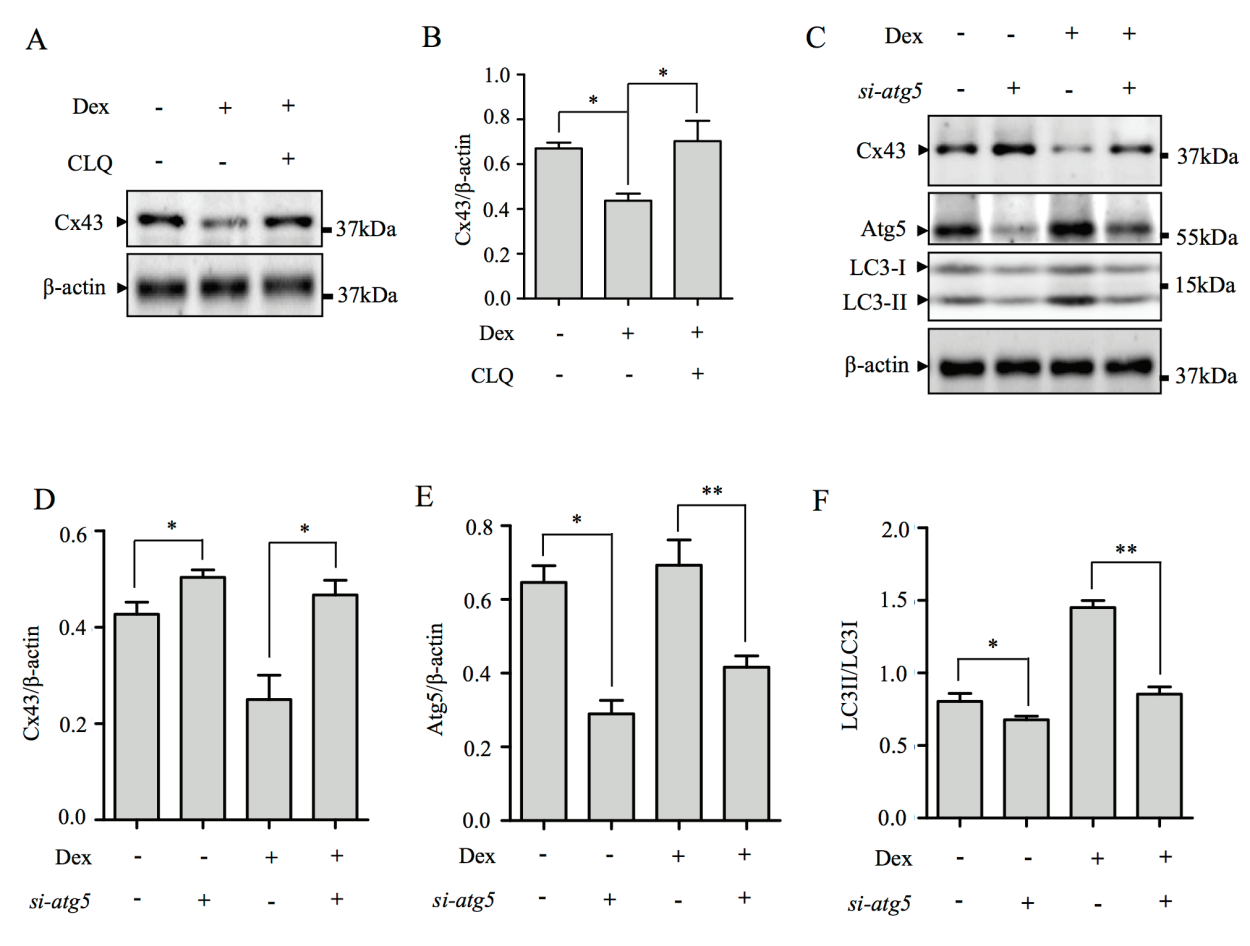
Cx43 is degraded by autophagy following Dex treatment (**A**) Inhibition of lysosome activity by CLQ blocked Dex-induced Cx43 degradation. Total cellular proteins (TCPs) from MLO-Y4 cells co-stimulated with 10^−6^M Dex and 50μM CLQ for 24hrs were immunoblotted with antibody against Cx43. β-actin served as loading and normalization control. (**B**) The protein levels of Cx43 were quantified and expressed as a ratio against β-actin. (**C**) Attenuation of autophagy by Atg5 silencing blocks Dex-induced Cx43 degradation. TCPs form Atg5 siRNA transfected MLO-Y4 cells with/without Dex treatment for 24hrs were immunoblotted with antibodies against Cx43, Atg5 and LC3-I/II. β-actin served as loading and normalization control. The protein levels Cx43 (**D**) and Atg5 (**E**), were quantified and expressed as a ratio against β-actin. (**F**) The protein levels of lipidated LC3-II were expressed as a ratio against LC3-I. All data presented are representative of at least three independent experiments. *p<0.05, **p<0.01.

To verify that Cx43 was being degraded *via* an autophagasomal/lysosomal pathway, Dex treated cells were co-treated with lysosomal inhibitor, chloroquine (CLQ), to block autophagasomal/lysosomal degradation processes. As shown in Figure 5A and 5B, treatment of cells with CLQ potently inhibited the decrease in Cx43 protein expression induced by Dex. Furthermore, inhibition of Dex-induced autophagy by Atg5 siRNA knockdown also attenuated the decrease in Cx43 protein expression (Figure 5C–5F). Silencing knockdown efficiency at 50nM was 48.7% reduction in Atg5 expression (Suppl. Figure 4). Together these data indicates that the decrease in Cx43 protein expression by Dex is likely due to Cx43 degradation *via* the autophagasomal/lysosomal degradative pathway.

### The Akt-mTORC1 signaling pathway is involved in GC-induced autophagy and Cx43 degradation

**Figure 6:**
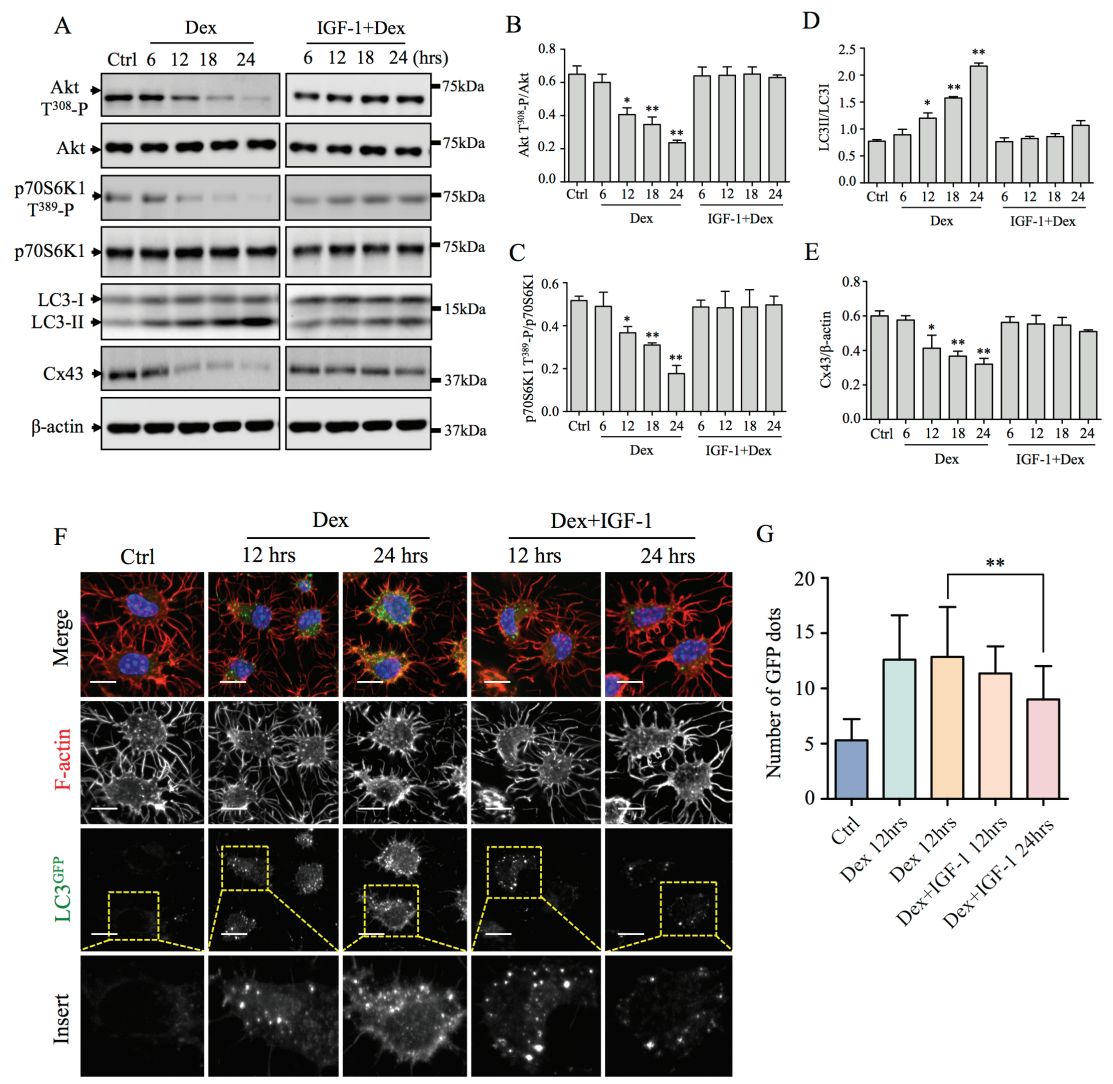
Akt-mTORC1 signaling pathway is involved in Dex-induced autophagy and Cx43 degradation (**A**) Dex inhibition of Akt and p70S6K phosphorylation, LC3 lipidation and Cx43 degradation were attenuated by the potent Akt activator, IGF-1. TCPs from MLO-Y4 cells treated with 10^−6^M Dex alone or in combination with 100nM IGF-1 for indicated times were immunoblotted with antibodies against p-Akt T^308^, total Akt, p-p70S6K1 T^389^, total p70S6K1, LC3 I/II, and Cx43. β-actin served as loading and normalization control. (**B**) The protein levels of phosphorylated Akt T^308^ were expressed as a ratio against total Akt. (**C**) The protein levels of phosphorylated p70S6K T^389^ were expressed as a ratio against total p70S6K. (**D**) The protein levels of lipidated LC3-II were expressed as a ratio against LC3-I. (**E**) The protein levels of Cx43 were expressed as a ratio against β-actin. (**F**) IGF-1 time-dependently suppressed and protected against Dex-induced autophagy *ex vivo.* Primary calvarial osteocytes from LC3^GFP^ mice treated with 10^−6^ M Dex or in combination with 100nM IGF-1 for 12hrs and 24hrs were fixed and immunostained with rhodamine-conjugated phalloidin (F-actin; red) and Hoechst 33258 (nuclei; blue). Immunoreactivity and fluorescence of LC3^GFP^ puncta were investigated by confocal microscopy. Scale bars = 10μm. (**G**) The average number of GFP puncta representing LC3^+ve^ autophagic vesicles were quantified. All data presented are representative of at least three independent experiments, with quantitative analysis calculated from at least 20 cells. Bar graph was compared to control and expressed as means ± SD. *P<0.05, **p<0.01.

The highly conserved Akt-mammalian target of rapamycin complex-1 (mTORC1) signaling hub, is widely considered to be the master inhibitor of the autophagic response [15]. Thus we also examined whether GC-induced autophagy was *via* the inhibition of Akt and mTORC1 signaling. As shown in Figure 6A–6E, Dex administration time-dependently inhibited the phosphorylation of Akt on T^308^ from 6hrs to 24hrs. Inhibition of Akt phosphorylation leads to the inactivation of downstream mTORC1 activity demonstrated by the time-dependent inhibition of classic mTORC1 substrate p70S6K1 phosphorylation on T^389^, an indicator of mTORC1 activity [16–18].

To provide further evidence of the involvement of Akt-mTORC1 signaling in GC-induced autophagy, we next used IGF-1, a potent Akt activator [19] to counteract the effects of Dex. As shown in Suppl. Figure 5A to 5C, IGF-1 alone can potently and time-dependently induced the phosphorylation of Akt on T^308^ and correspondingly, the phosphorylation of p70S6K1 on T^389^ in MLO-Y4 cells *in vitro*. More importantly, IGF-1 mitigated the inhibitory effect of Dex on Akt and p70S6K1 phosphorylation. This attenuation of the inhibitory effect of Dex on Akt-mTORC1 signaling was also found to sufficiently negate the autophagic degradation of Cx43 induced by Dex (Figure 6A). Confocal immunofluorescence analysis of Dex and IGF-1 co-stimulated primary calvarial osteocytes from LC3^GFP^ mice demonstrated reduced autophagy according to decreased number of LC3^GFP^ puncta as compared to Dex alone treatment (Figure 6F and 6G). Collectively, these results demonstrate that the inhibition of the Akt-mTORC1 signaling by GC is responsible for inducing autophagy and the subsequent degradation of Cx43. Reactivation of this pathways for example using IGF-1, is sufficient to negate this effect and may offer protection against the deleterious effect of GC on osteocyte connectivity.

## DISCUSSION

Using complementary *ex vivo* primary calavarial osteocyte cultures and *in vitro* biochemical analyses of MLO-Y4 osteocyte-like cells, we showed for the first time that GC-induced autophagy is responsible for the degradation of Cx43. The degradation of Cx43 was found to be associated with shortening of dendritic processes and loss of dendritic connectivity, leading to disruption of intercellular communication between osteocytes. Inhibition of the Akt-mTORC1 signaling hub was found to mediate the effects of GC-induced autophagy and subsequent Cx43 degradation. Conversely, re-activation of Akt-mTORC1 pathway by IGF-1 protects against GC-mediated Cx43 degradation.

Cx43 is the most abundant hemichannels protein expressed in osteocytes. It serves to mediate intercellular communications between adjacent neighboring osteocytes when two opposing Cx43 hemichannels localized on dendritic tips forms a gap junction [3]. In addition, unpaired hemichannels allow for communication between embedded osteocytes with the extracellular milieu or with cells on the bone surface, such as with osteoblasts and osteoclasts [3]. In this study, we demonstrated that GC treatment in osteocytes causes the shortening of dendritic tip/plasma membrane and internalization Cx43 to cytoplasmic autophagosomal/lysosomal degradative vesicles leading to a marked reduction in the total protein expression of Cx43. Both lysosomal inhibition with CLQ and RNAi-mediated knockdown of Atg5, a key autophagic protein resulted in attenuation of Cx43 degradation, indicating that the blockade of either GC-induced autophagy or the autophagic degradative process preserves Cx43 expression and osteocyte connectivity.

Interestingly, it was recently reported that under basal conditions, plasma membrane (PM) Cx43 inhibits autophagy and prevents unnecessary loss of PM to autophagosome biogenesis [20]. However upon induction of autophagy, PM Cx43 is internalized into newly formed autophagosomes and subsequently degraded in mature autolysosomes. The degradation of Cx43 by autophagy leads to sustained activation of autophagy. Thus in osteocytes, under basal conditions, Cx43-dependent inhibition of autophagy may help in maintaining the dendritic morphological appearance of the osteocyte by preventing unnecessary loss of PM to autophagosome biogenesis. However, upon GC administration and subsequent induction of autophagy, this inhibitory signal is lost leading to parts of the PMs being redirected towards autophagosome biogenesis resulting in the observed shortening of the osteocyte dendritic processes. At the same time, Cx43 localized at these sites are incorporated into autophagosome which consequently results in its subsequent degradation. Our data showed that autophagy coordinates the events of osteocyte dendritic shortening, PM Cx43 internalization and degradation.

The highly conserved mammalian target of rapamycin complex-1 (mTORC1) and its associated signaling pathway is widely considered to be the master negative regulator of the autophagy [15]. In our study, we demonstrated that GC-induced autophagy is dependent on the inhibition of Akt-mTORC1 signaling. Furthermore, reactivation of this pathway with the Akt-mTORC1 activator, IGF-1, was found to prevent dephosphorylation of Akt and S6K1 induced by GC resulting in the attenuation of autophagy and subsequent degradation of Cx43.

In conclusion, our study has shown for the first time that GC in the form of Dex triggers Cx43 degradation and shortening of dendritic processes *via* the induction of autophagy in osteocytes. Although further studies are required, our data suggest that the Akt-mTORC1 signaling pathway plays an important role in GC-induced autophagy in osteocytes. This study provides further evidence on the deleterious effect of GC on osteocyte biology which, in part, may account for the severe adverse skeletal effects associated with therapeutic GC administration.

## MATERIALS AND METHODS

### Animals

9.6kb Dmp1^Cre^ transgenic mice were kindly provided by Prof. Jian Q. (Jerry) Feng at Texas A&M University Baylor College of Dentistry, USA [21]. The global mT/mG transgenic mice were purchased from Model Animal Research Center of Nanjing University, and were crossed with the Dmp1^Cre^ transgenic mice to make osteocyte-specific Dmp1^Cre^-mT/mG transgenic mice at Zhejiang Academy of Medical Sciences under Animal Ethics Approval Number SYXK (Zhe) 2014-0008. The mT/mG double fluorescent reporter mice expresses strong membrane-targeted Tomato (mT: red fluorescence) in all tissues prior to Cre excision and membrane targeted green fluorescence (mG: EGFP green fluorescence) following Cre excision in cells that specifically expresses the Cre recombinase, i.e. only in osteocytes in our Dmp1^Cre^-mT/mG mice. The global LC3^GFP^ transgenic mice originally generated by Noboru Mizushima, Department of Physiology and Cell Biology, Tokyo Medical and Dental University, Japan [22] were kindly provided by Prof. Yeguang Chen, School of Life Sciences, Tsinghua University, China. Mice were housed under specific pathogen free (SPF) conditions in Makrolon type II cages, at environmental temperature of 20-22°C, relative humidity 50-70%, and under a 12hrs day/night light cycle. They were fed a commercial diet from Shanghai Pu Lu Teng Biological Technology Co. Ltd. and provided with tap water *ad libitum*. Sixteen male 1-week old C57/BL6 Dmp1^Cre^-mT/mG transgenic mice and twenty male 1-week old C57/BL6 global LC3^GFP^ transgenic mice were euthanized and calvariae dissected into 3mm X 3mm squares and fixed in 4% paraformaldehyde (PFA) for 2hrs at room temperature (RT). Calvariae from Dmp1^Cre^-mT/mG transgenic mice were randomly divided into four treatment groups: no treatment control and 10^−6^M Dex for 2, 12, and 24hrs. Calvariae from LC3^GFP^ transgenic mice were randomly divided into five treatment groups: no treatment control, 10^−6^M Dex treatment for 12 and 24hrs, and co-treatment with 10^−6^M Dex and 100nM IGF-1 for 12 and 24hrs. All treatment groups displayed normal behavioral activity throughout the experimental period and no adverse effects or fatalities were observed. All animal care and experimental procedures conducted in this study complied with the guidelines for Ethical Conduct in the Care and Use of Non-human Animals in Research by the American Psychological Association and were approved by the Animal Care and Experiment Committee of Zhejiang Academy of Medical Sciences.

### Reagents and antibodies

For immunostaining, Premo^TM^ Autophagy Tandem Sensor RFP-GFP-LC3B, Hoechst 33258, Rhodamine-conjugated Phalloidin, and Alexa Fluor^®^ 568 and Alexa Fluor^®^ 647 goat anti-Rabbit IgG (H+L) secondary antibodies were from Molecular Probes, Life Technologies^®^, USA. CYTO-ID^®^ Autophagy Detection Kit was from Enzo Life Sciences, USA. LysoTracker^®^ Red DND-99 was from Thermo Fisher Scientific, USA. Rab7-GFP plasmid was kindly provided by A/Prof. Nathan J. Pavlos from the University of Western Australia. For western blot analysis, anti-β-actin JLA20 was from Developmental Studies Hybridoma Bank (DSHB); anti-Cx43, anti-LC3 A/B, anti-p-Akt T^308^, anti-Akt, anti-p-p70S6K1 T^389^ and anti-p70S6K1 were from Cell Signaling Technology^®^, USA. Dexamethasone (Dex) and Insulin-like Growth Factor-1 (IGF-1) human was from Sigma-Aldrich^®^, USA. Chloroquine (CLQ) was from Invitrogen^®^, USA.

### Cell culture

For *in vitro* culture, MLO-Y4 cells were maintained in α-MEM supplemented with 5% fetal bovine serum (FBS) and 5% fetal calf serum (FCS), 1% penicillin and streptomycin in 37°C humidified 5% CO_2_ incubator. Cells were plated on type-I collagen coated plates or coverslips and propagated to 70% confluence prior to experimental procedures. For dose-dependent experiments, cells were treated with varying concentrations (10^−8^M to 10^−3^M) of Dex. For time-dependent experiments, cells were treated with 10^−6^M Dex for various durations of time. For *ex vivo* culture, calvariae from 5-day old Dmp1^Cre^-mT/mG mice or LC3^GFP^ mice were washed three times with phosphate-buffered saline (PBS) followed by three washes with α-MEM containing 1% penicillin and streptomycin. The periosteum was then gently stripped off from both sides, and the calvariae were maintained in α-MEM supplemented with 5% FBS and 5% FCS, 1% penicillin and streptomycin at 37°C humidifed 5% CO_2_ incubator. Dose- and time-dependent experiments were carried out as described above.

### Transfection

MLO-Y4 cells (1×10^5^/well) were plated in type-I collagen coated six-well culture plates, and then transfected with various concentrations (12.5nM, 25nM, 50nM and 100nM) of Atg5 siRNA oligo cocktail (Stealth siRNAs: MSS247019, MSS247020, MSS247021, from Thermo Fisher^®^, USA) or negative control using Lipofectamine^®^ 3000 Transfection Reagent (Invitrogen^®^, USA) following the manufacturer's protocol. The cells were harvested 24hrs after transfection.

### Confocal microscopy

MLO-Y4 cells cultured on glass coverslips and calvarial primary osteocytes *ex vivo* cultured in 96-wells plates were fixed for 15mins and and 2hrs at RT with 4% PFA respectively. Cells were then permeabilized for 5 minutes with 0.1% Triton X-100 in PBS, and non-specific antibody binding was blocked by 3% BSA-PBS for 30mins. MLO-Y4 cells and primary calvarial osteocytes were incubated with primary antibody diluted in 0.2% BSA-PBS for 2hrs at RT or overnight at 4°C respectively. Following incubation cells were washed extensively and bound primary antibodies were reacted with fluorescence conjugated secondary antibody for 1hr at RT, and nuclei were stained with Hoechst 33258 dye for 15mins at RT. Coverslips were then mounted in ProLong Diamond Anti-fade medium (Invitrogen, USA). Images were acquired on a Nikon A1 confocal microscope equipped with 60X (oil immersion) lens, and analyzed using ImageJ software. For quantification of the number and length of dendritic processes and the number of Cx43 fluorescence dots and endogenous LC3^GFP^ punctual of each osteocyte, the channels were extracted and converted to greyscale images and analyzed using ImageJ software.

### Western blot analysis

Total cellular proteins (TCP) were extracted using RIPA lysis buffer (150mM NaCl, 50mM Tris-HCl pH 8.0, 1.0% NP40, 0.5% sodium deoxycholate and 0.1% SDS) containing protease inhibitor cocktail (Roche) and phosphatase inhibitor cocktails (Sigma-Aldrich^®^, USA) for 30mins on ice. Cellular debris were clarified by centrifugation at 16,000g for 20mins at 4°C. Cleared TCP were diluted with 4X SDS sampling buffer and boiled for 5mins. 30μg of TCP were separated on 10%-17.5% SDS-PAGE gel and resolved protein transferred to nitrocellulose membrane for analysis by western blot. Membranes were blocked with 5% skim milk in TBS-Tween (TBST) for 1hr and then incubate with primary antibodies diluted in 1% (w/v) skim milk in TBST for 2hrs shaking at RT or overnight shaking at 4°C. Membranes were washed three times with TBST and then incubated with the corresponding HRP-conjugated secondary antibodies. After three times TBST and three times TBS washes, antibody reactivity were detected using the Western Lightning Ultra Detection Kit (Perkin Elmer, USA) using the FujiFilm LAS-3000/4000 Gel Documentation System (Japan) and its associated software. Immunoblot images presented are representatives of at least three independent experiments.

### LDH cytotoxicity assay

The Lactate Dehydrogenase (LDH) Cytotoxicity Assay Kit (BioVision, Mountain View, CA, USA) was used for quantification of plasma membrane damage. MLO-Y4 cells were seeded in a 96-well plate overnight at 37°C. After treatment with Dex for 24hrs, the plates were centrifuged at 250g for 10mins and 100μl of supernatant was transferred into corresponding wells of a clear 96-well plate. To the supernatant, 100μl of reaction mixture was added to each well and incubated for 30mins in the dark. The absorbance at 490nm was then measured using a spectrophotometer microplate reader BioRAD spectrophotometer (Model 680). As positive control, the maximum release of LDH was determined by incubation of the cells in 1% Triton X-100 for 1hr in culture medium.

### Analysis of apoptosis by flow cytometry

MLO-Y4 cells were treated with Dex (10^−6^M and 10^−3^M) for 24hrs and then suspended in binding buffer and stained with Annexin V-PE and 7-amino-actinomycin (7-AAD) for 15mins at RT in the dark. Cells were excited at 488nm and signals from 10,000 cells acquired at 585/42 (564 to 606nm) and 702/64 (670 to 735nm) in a FACS Canto II (BD). Results were analyzed using the FACSDiva (BD) software and expressed as the percentage of apoptotic cells within each population.

### Statistical analysis

Comparisons of individual data sets were performed in most experimental procedure using Student's *t*-test, and data are presented as the Mean ± SD. *p*-values < 0.05 was considered statistically significant.

## SUPPLEMENTARY MATERIALS FIGURES


