# Correction: Glucocorticoid impairs cell-cell communication by autophagy-mediated degradation of connexin 43 in osteocytes

**DOI:** 10.18632/oncotarget.27035

**Published:** 2019-06-18

**Authors:** Junjie Gao, Tak Sum Cheng, An Qin, Nathan J. Pavlos, Tao Wang, Kai Song, Yan Wang, Lianzhi Chen, Lin Zhou, Qing Jiang, Hiroshi Takayanagi, Sheng Yan, Minghao Zheng

**Affiliations:** ^1^ Centre for Orthopaedic Research, School of Surgery, The University of Western Australia, Nedlands, Western Australia, Australia; ^2^ Shanghai Key Laboratory of Orthopedic Implants, Department of Orthopedic Surgery, Shanghai Ninth People’s Hospital, Shanghai Jiao Tong University School of Medicine, Shanghai, China; ^3^ Division of Orthopaedic Surgery, Department of Surgery, Guangdong General Hospital, Guangdong Academy of Medical Sciences, Guangzhou, China; ^4^ Key Laboratory of Combined Muti-organ Transplantation, Ministry of Public Health, State Key Laboratory for Diagnosis and Treatment of Infectious Diseases, Division of Hepatobiliary Pancreatic Surgery, First Affiliated Hospital, Zhejiang University School of Medicine, Hangzhou, China; ^5^ Department of Sports Medicine and Adult Reconstruction Surgery, Drum Tower Hospital Affiliated to Medical School of Nanjing University, Nanjing, Jiangsu, China; ^6^ Department of Immunology, Graduate School of Medicine and Faculty of Medicine, The University of Tokyo, Tokyo, Japan; ^7^ State Key Laboratory for Diagnosis and Treatment of Infectious Diseases, Collaborative Innovation Center for Diagnosis and Treatment of Infectious Diseases, The First Affiliated Hospital, College of Medicine, Zhejiang University, Hangzhou, China

**This article has been corrected:** Due to a software malfunction while cropping the western blot image into the PDF, the previous image for β-action (in IGF-1+Dex group) and for Akt T^308^ -P (in Dex group) in Figure 6A is incorrect. The corrected Figure 6A is shown below. The original image for western blot is also attached. The authors declare that these corrections do not change the results or conclusions of this paper.

Original article: Oncotarget. 2016; 7:26966–26978. 26966-26978 . https://doi.org/10.18632/oncotarget.9034

**Figure 6:**
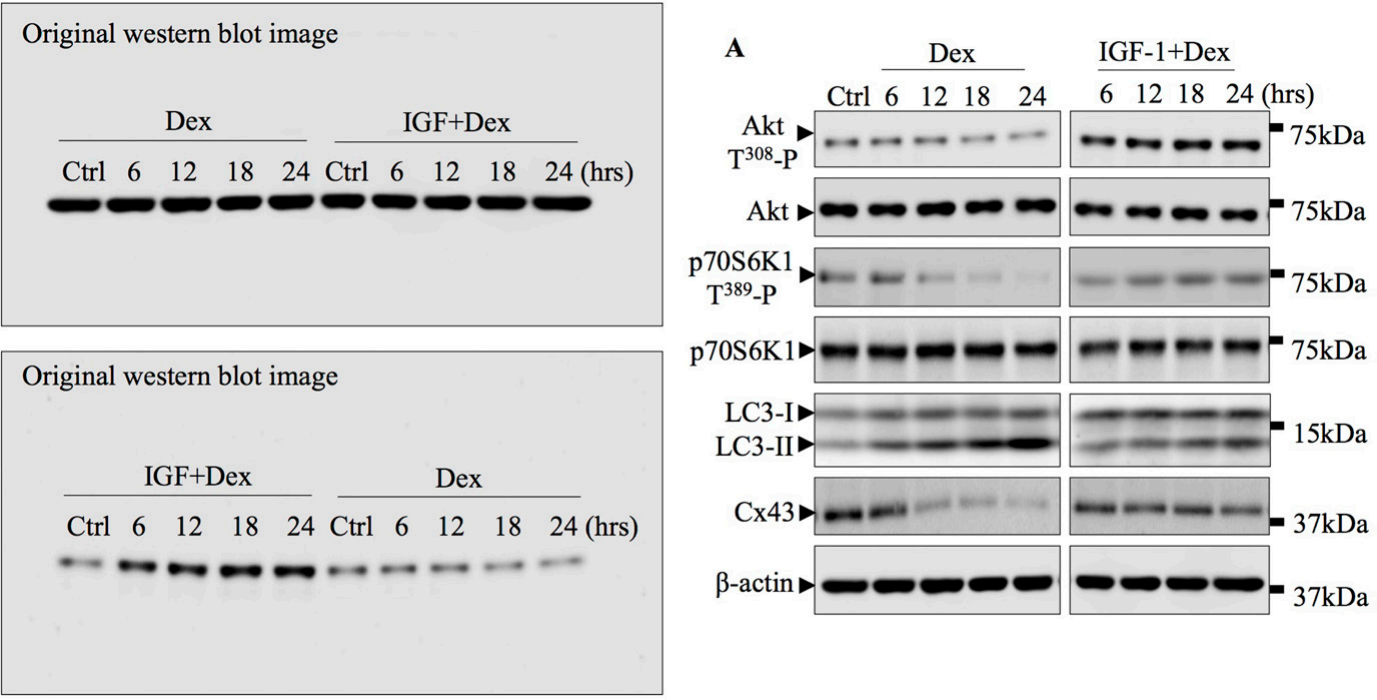
Akt-mTORC1 signaling pathway is involved in Dex-induced autophagy and Cx43 degradation. (A) Dex inhibition of Akt and p70S6K phosphorylation, LC3 lipidation and Cx43 degradation were attenuated by the potent Akt activator, IGF-1. TCPs from MLO-Y4 cells treated with 10-6M Dex alone or in combination with 100nM IGF-1 for indicated times were immunoblotted with antibodies against p-Akt T308, total Akt, p-p70S6K1 T389, total p70S6K1, LC3 I/II, and Cx43. β-actin served as loading and normalization control.

