# Supplementary material for: Glucocorticoid impairs cell-cell communication by autophagy-mediated degradation of connexin 43 in osteocytes
Source: Oncotarget. 2016 Apr 27;7(19):26966–78. doi: 10.18632/oncotarget.9034 (PMC5053625; doi:10.18632/oncotarget.9034)
Supplement: Supplementary file 1 [file oncotarget-07-26966-s001.pdf]

Glucocorticoid impairs cell-cell communication by autophagy-mediated degradation of connexin 43 in osteocytes

Supplementary Material

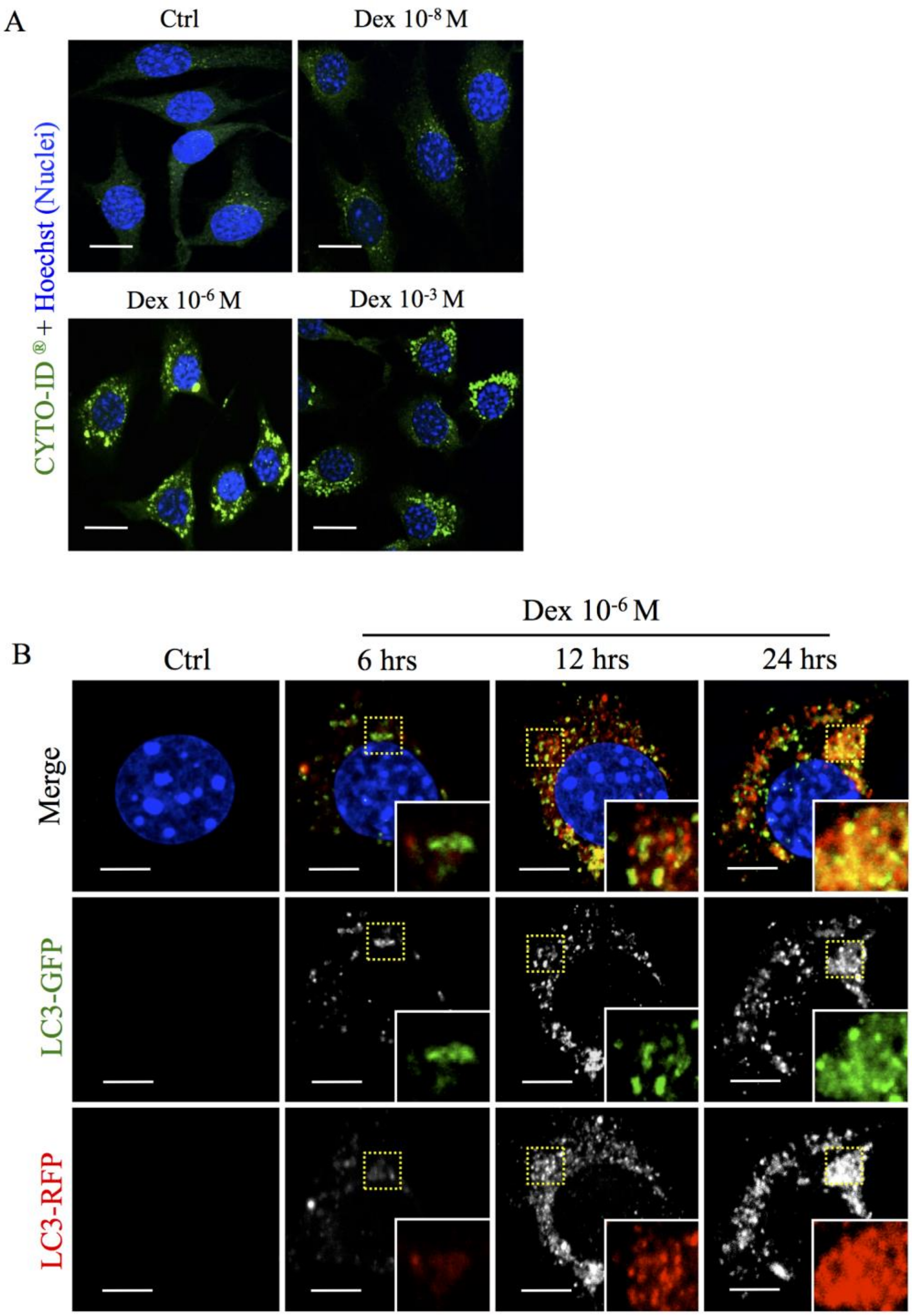

Supplemental Figure 1. Dex dose- and time-dependently induced autophagy in MLO-Y4 cells *in vitro*. **(A)** MLO-Y4 cells treated with indicated doses of Dex for 24hrs were fixed and stained with CYTO-ID® (LC3; green) and Hoechst 33258 (nuclei; blue). Immunoreactivities

were visualized under confocal microscopy. Scale bars = 10μm. **(B)** Dex time-dependently induces autophagic flux and the progressive conversion and maturation of neutral autophagosomes (green) to acidic autolysosomes (red) in MLO-Y4 cells *in vitro*. MLO-Y4 cells transiently transfected with tandem autophagy sensor RFP-GFP-LC3B and treated with 10^{-6}M Dex for indicated time were fixed and autophagic flux were examined by confocal microscopy. Scale bars = 5μm.

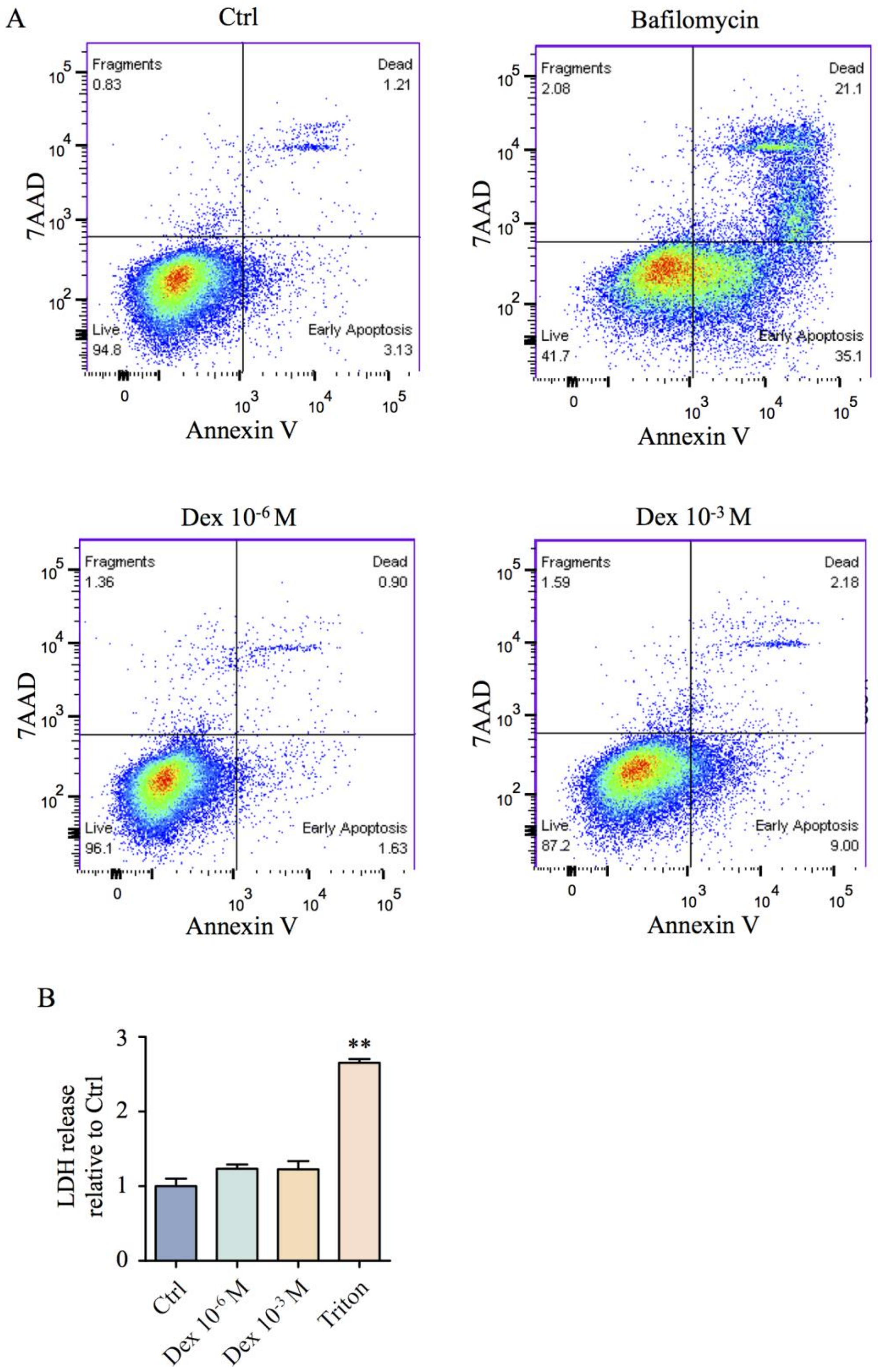

Supplemental Figure 2. Dex did not induce apoptosis in MLO-Y4 cells under conditions in which autophagy was induced. **(A)** MLO-Y4 cells treated with 10^{-6} and 10^{-3}M Dex for 24hrs were stained with Annexin V-PE and 7-AAD and apoptosis analysed by FACS. Results were

expressed as the percentage of dead and early apoptotic cells within each population. Compared to positive control, bafilomycin A1, which induces apoptosis in 56.2% cells (% dead cells + % early apoptosis cells), Dex only induced apoptosis in 2.53% and 11.18% of total cell population following 24hrs of 10^{-6}M and 10^{-3}M Dex treatment respectively. **(B)** Dex do not affect MLO-Y4 cells membrane integrity. After 24hrs treatment with indicated doses of Dex, plasma membrane damage and necrosis were examined by the release of lactate dehydrogenase (LDH) activity. Triton-X100 treated MLO-Y4 cells were used as positive membrane damage control group. All data presented are representative of at least three independent experiments, with quantitative analysis calculated from at least 20 cells. All bar graphs were compared to control with **p<0.01 for statistical significance.

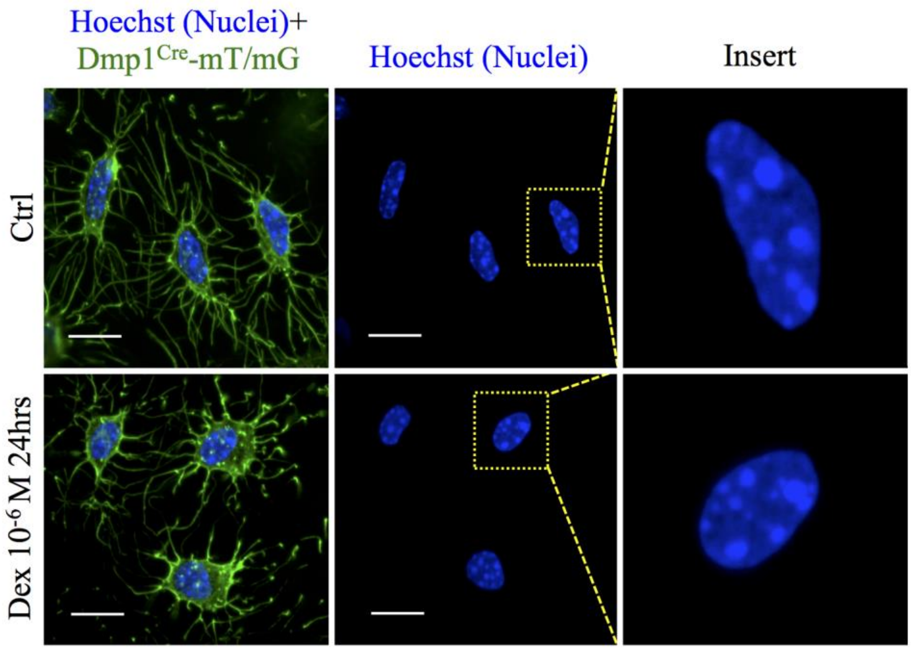

Supplemental Figure 3. Dex did not affect nuclei integrity in *ex vivo* cultures of primary calvarial osteocytes from DMP1^{Cre}-mT/mG mice. Following 24hrs treatment with 10^{-6}M Dex, calvarium were fixed, stained with Hoechst 33258 and visualized under confocal microscopy. Scale bars = 10μm.

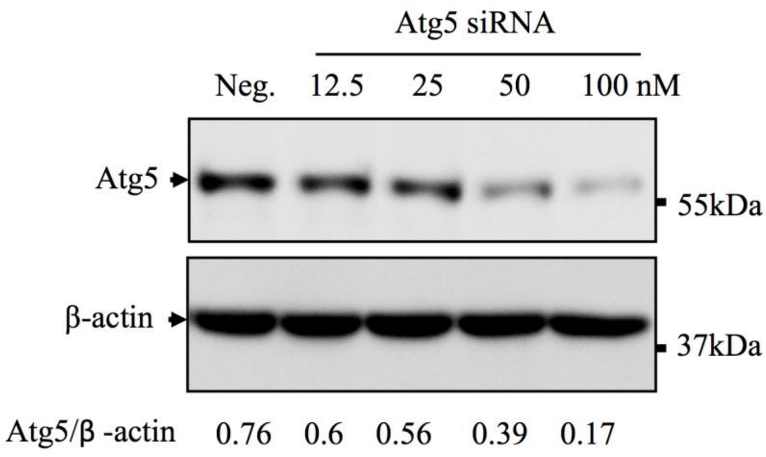

Supplemental Figure 4. Atg5 siRNA oligos efficiently inhibited Atg5 protein expression. TCPs from MLO-Y4 cells transfected with cocktail of Atg5 siRNA oligos or negative control at indicated concentrations for 24hrs were subjected to western blot analysis using specific antibody against Atg5. Antibody against β-actin serves as internal loading and normalization control. The protein levels of Atg5 were expressed as a ratio against β-actin.

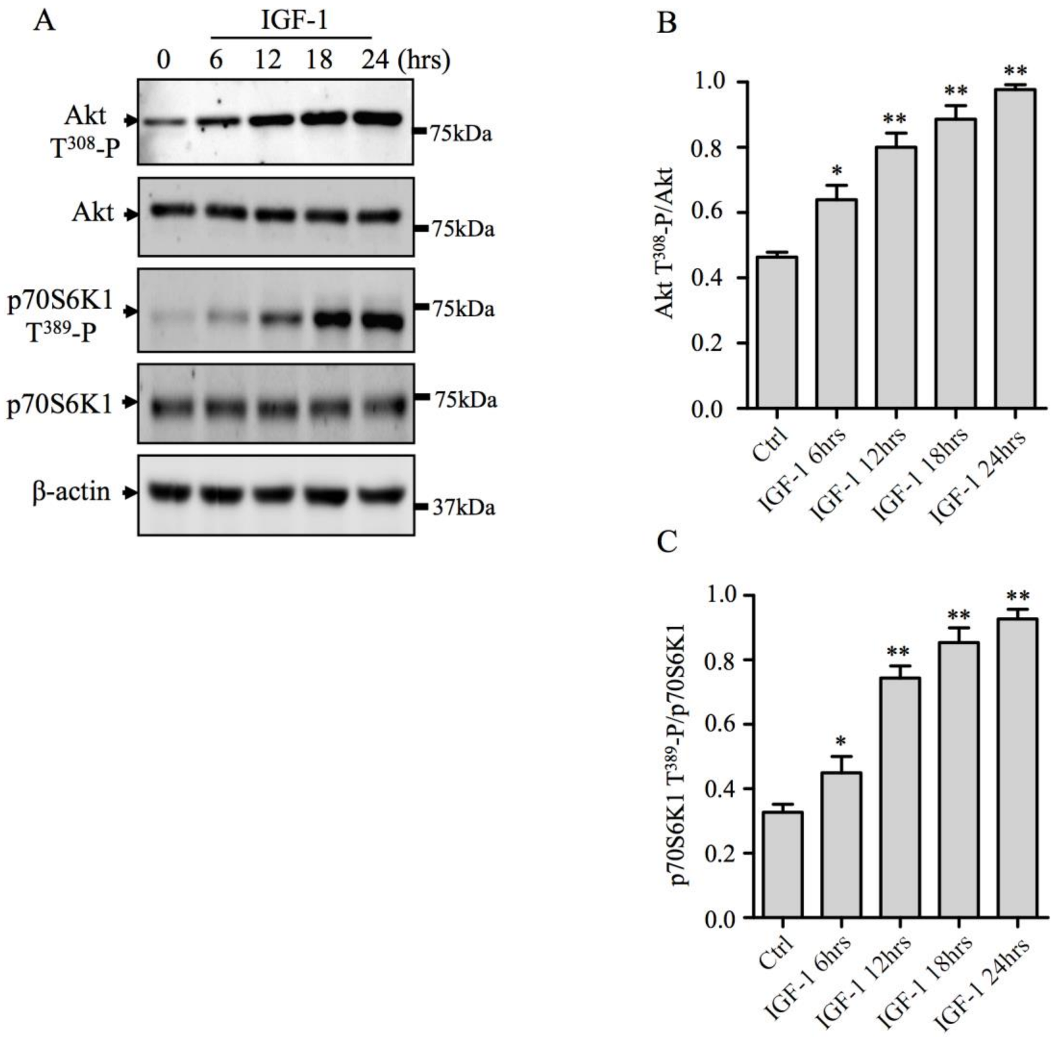

Supplemental Figure 5. IGF-1 alone potently induced Akt and p70S6K1 phosphorylation in MLO-Y4 cells in a time-dependent manner. **(A)** TCPs from MLO-Y4 cells treated with 100nM IGF-1 for indicated duration of time were immunoblotted using specific antibodies against both phosphorylated and total forms of Akt and p70S6K1. β-actin served as loading and normalization control. **(B)** The protein levels of phosphorylated Akt T^{308} were expressed as a ratio against total Akt. **(C)** The protein levels of phosphorylated p70S6K T^{389} were expressed as a ratio against total p70S6K. All data presented are representative of at least three independent experiments. *p<0.05, **p<0.01.
